# Perinephric Hematoma Following Extracorporeal Shock Wave Lithotripsy: A Comprehensive Review of Four Decades of Evidence on Risk Factors, Prevention, and Management

**DOI:** 10.7759/cureus.94565

**Published:** 2025-10-14

**Authors:** Ahmed Abdelrasheed, Muhammad Iqbal

**Affiliations:** 1 Urology, Royal Glamorgan Hospital, Pontyclun, GBR; 2 Urology, Cwm Taf University Health Board, Royal Glamorgan Hospital Llantrisant, Cardiff, GBR

**Keywords:** eswl complications, extracorporeal shock wave lithotripsy, kidney stones, perinephric hematoma, renal hemorrhage

## Abstract

Perinephric hematoma represents a significant complication following extracorporeal shock wave lithotripsy (ESWL), with reported incidence ranging from 0.1% to 30% depending on detection methodology. This comprehensive review examines four decades of evidence since the first clinical ESWL treatment in 1980, analyzing risk factors, prevention strategies, and management approaches. Following a systematic literature search, we identified 36 relevant studies encompassing 30,347 treated patients. Hypertension emerges as the dominant modifiable risk factor, increasing hematoma rates from a baseline of 0.66% to 3.8% in poorly controlled patients. Stepwise voltage ramping protocols demonstrate remarkable protective effects, reducing incidence by over 50%. Conservative management succeeds in over 95% of cases, with arterial embolization reserved for active bleeding. Paradoxically, the original Dornier HM3 lithotripter maintains superior safety profiles compared to modern devices despite technological advances. Long-term follow-up studies document complete renal function preservation following appropriate management. These findings inform evidence-based protocols optimizing safety while maintaining treatment efficacy.

## Introduction and background

Extracorporeal shock wave lithotripsy (ESWL) revolutionized urolithiasis management following its introduction by Christian Chaussy in Munich on February 7, 1980 [1]. This non-invasive technique uses focused acoustic energy to fragment kidney stones, enabling spontaneous passage of smaller fragments [2]. Despite remarkable success in treating millions of patients worldwide, ESWL carries inherent risks of renal hemorrhage ranging from subclinical hematomas to life-threatening bleeding [3].

The mechanism of ESWL-induced renal injury involves complex interactions between shock wave physics and tissue biomechanics. High-energy acoustic waves generated outside the body focus on targeted stones, creating pressure gradients exceeding 100 MPa [4]. These forces fragment calculi through direct stress waves and cavitation bubble collapse but inevitably affect the surrounding renal parenchyma and vasculature [5]. Hemorrhagic complications result from direct vascular injury, with severity correlating to total energy delivered, shock wave characteristics, and patient-specific factors [6].

The initial optimism regarding ESWL safety proved premature. Chaussy's pioneering 1982 report claimed "no complications have resulted from tissue exposure to high-energy shock waves" after treating 72 patients [1]. However, subsequent larger series revealed that perinephric hematomas were the most significant acute complication, occurring in 0.1-30% of patients, depending on detection methods and definitions [7,8]. This wide incidence range reflects evolution in imaging capabilities, with routine cross-sectional imaging detecting many asymptomatic hematomas missed by clinical observation alone [9].

The clinical significance of post-ESWL hematomas varies dramatically. While most remain asymptomatic and resolve spontaneously, severe cases may present with hemodynamic instability, require transfusion, or necessitate interventional procedures [10]. Understanding risk factors, implementing prevention strategies, and optimizing management approaches remain critical for maximizing ESWL safety. This comprehensive review synthesizes four decades of evidence to guide contemporary clinical practice.

## Review

Methods

This systematic literature review followed the Preferred Reporting Items for Systematic Reviews and Meta-Analyses (PRISMA) 2020 guidelines [11]. Database searches included PubMed/MEDLINE, EMBASE, Cochrane Library, Web of Science, and Google Scholar from January 1980 through December 2024. Search terms combined: "extracorporeal shock wave lithotripsy" OR "ESWL" OR "SWL" AND "hematoma" OR "hemorrhage" OR "bleeding" OR "complications" AND "kidney" OR "renal" OR "perinephric". Reference lists of identified articles were manually reviewed for additional relevant studies.

Inclusion criteria encompassed (1) studies reporting perinephric hematoma incidence, risk factors, or management after ESWL; (2) English language full-text availability; (3) human subjects; and (4) peer-reviewed publication. Exclusion criteria included (1) case reports with fewer than 10 patients unless providing unique clinical insights; (2) studies focusing exclusively on non-renal ESWL applications; (3) abstracts without full publication; and (4) duplicate patient cohorts.

**Figure 1:**
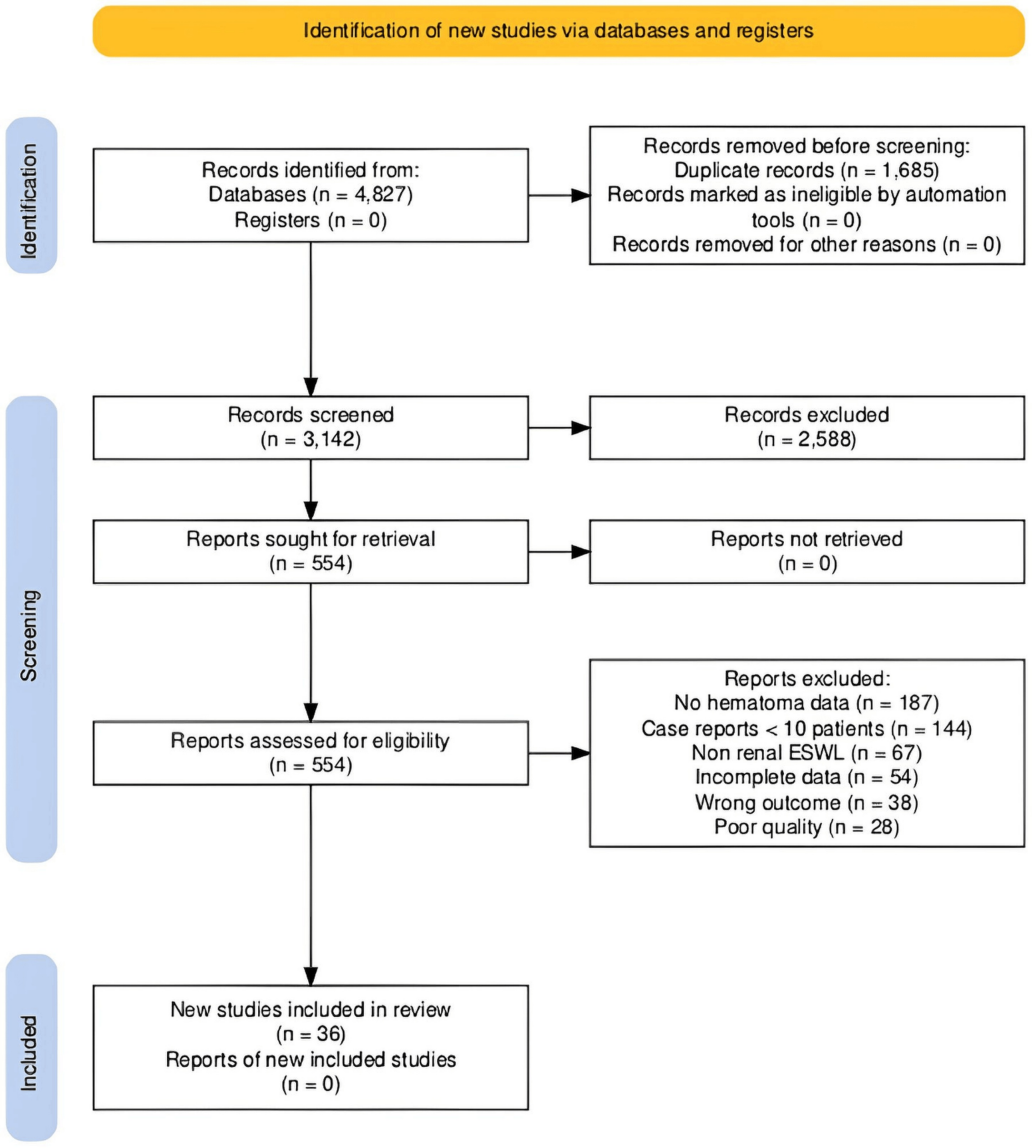
PRISMA 2020 Flow Diagram Source: [11]

The study selection process is detailed in Figure 1 (PRISMA flow diagram), which illustrates the systematic screening from 4,827 initially identified records to the final inclusion of 36 studies encompassing 30,347 patients (Table 1).

**Table 1:** Summary of All Included Studies (n=36) NOS: Newcastle-Ottawa Scale; RoB: Risk of Bias Tool; ESWL: Extracorporeal Shock Wave Lithotripsy; CT: Computed Tomography; US: Ultrasound; MRI: Magnetic Resonance Imaging; BMI: Body Mass Index

Ref	Study	Year	Study Design	Sample Size	Country	Lithotripter Type	Detection Method	Hematoma Rate (%)	Main Focus	Quality Score (NOS/RoB)
[1]	Chaussy et al.	1980	Prospective cohort	72	Germany	Dornier HM3	Clinical	0	Initial ESWL experience	7/9
[5]	Evan et al.	1998	Experimental	125	USA	Dornier HM3	MRI	2.4	Renal trauma mechanisms	7/9
[7]	Dhar et al.	2004	Retrospective cohort	318	USA	Dornier Doli	CT selective	3.5	Risk factors multivariate	8/9
[8]	Knapp et al.	1996	Retrospective cohort	3,620	Austria	Dornier HM3	Clinical	0.66	Blood pressure & resistive index	8/9
[9]	Wong et al.	2024	Prospective cohort	573	China	Dornier Compact Delta	CT routine	30.9	Comprehensive risk factors	9/9
[10]	Razvi et al.	2012	Case-control	1,010	Canada	Dornier Doli	CT selective	2.8	Risk factors matched analysis	8/9
[14]	Salem	2009	RCT	475	Egypt	Dornier SLX	Clinical	3.6	ESWL vs. ureteroscopy	Unclear RoB
[15]	Moschetta et al.	2016	Prospective cohort	478	Italy	Storz Modulith	CT routine	28.9	CT diagnostic/prognostic role	8/9
[16]	Newman et al.	1986	Retrospective cohort	238	USA	Dornier HM3	Clinical	0.4	Pediatric ESWL experience	7/9
[17]	Kostakopoulos et al.	1995	Retrospective cohort	4,247	Greece	Dornier HM3	Clinical/US	0.66	Risk factors landmark study	8/9
[18]	Zanetti et al.	1999	Prospective cohort	822	Italy	Various	Clinical	1.2	Cardiac dysrhythmias	7/9
[19]	Eaton et al.	2003	Prospective cohort	156	USA	Dornier Compact	Clinical	1.9	Serum troponin levels	7/9
[20]	Carey et al.	1992	Retrospective cohort	28	USA	Dornier HM3	Clinical	3.6	Anticoagulation with aneurysms	6/9
[21]	Rosenberg et al.	2011	Retrospective cohort	445	USA	Dornier Compact	Clinical	2.5	Antiplatelet therapy safety	7/9
[22]	Ng et al.	2012	Case-control	367	Hong Kong	Dornier Compact	CT selective	3.0	Hepatic hematoma, energy	8/9
[23]	El-Nahas et al.	2007	Prospective cohort	384	Egypt	Siemens Lithostar	CT	1.8	Stone disintegration predictors	8/9
[24]	Logarakis et al.	2000	Retrospective cohort	463	Canada	Dornier HM3	Clinical/US	1.3	Clinical outcome variation	7/9
[25]	Nussberger et al.	2017	Retrospective cohort	868	Switzerland	Storz Modulith	CT routine	15.4	BMI extremes as a risk factor	8/9
[26]	Abdel-Khalek et al.	2004	Prospective cohort	620	Egypt	Dornier MFL 5000	Clinical/US	0.8	Success rate prediction model	8/9
[27]	Krishnamurthi et al.	1995	Retrospective cohort	620	USA	Dornier HM3	CT	1.6	Long-term outcomes	8/9
[28]	Gerber et al.	2005	Retrospective cohort	3,065	Switzerland	HM3/Modulith/Lithoskop	Clinical	0.66-3.0	Three-generation comparison	9/9
[29]	Zehnder et al.	2011	RCT	1,556	Switzerland	Modified HM3/Modulith SLX	CT selective	1.0-3.0	HM3 vs. Modulith RCT	Low RoB
[30]	Tailly	2002	RCT	1,156	Belgium	Storz Modulith SLX	Clinical	2.7	Electrohydraulic vs. electromagnetic	Low RoB
[31]	Graber et al.	2003	RCT	877	Switzerland	Siemens Lithoskop	CT	2.1	Two lithotriptor comparisons	Low RoB
[32]	Köhrmann et al.	1995	Prospective cohort	865	Germany	Modulith SL 20	Clinical/US	1.8	Third-generation introduction	7/9
[33]	Sheir et al.	2008	RCT	1,801	Egypt	Dornier Compact S	Clinical/US	2.5	Twin-pulse technique	Low RoB
[34]	Collado Serra et al.	1999	Retrospective cohort	559	Spain	Dornier MPL 9000	CT	3.1	Hematoma management	7/9
[35]	Newman et al.	1991	Retrospective cohort	412	USA	Dornier HM3	Ultrasound	0.5	Risk factors identification	7/9
[36]	Rubin et al.	1987	Prospective cohort	150	USA	Dornier HM3	CT	13.3	CT evaluation post-ESWL	8/9
[37]	Kaude et al.	1985	Prospective cohort	246	USA	Dornier HM3	CT	2.4	Renal morphology & function	8/9
[38]	Skuginna et al.	2016	RCT	289	Switzerland	Storz Modulith SLX	CT routine	5.6-13.0	Voltage ramping protection	Low RoB
[39]	Yilmaz et al.	2005	RCT	726	Turkey	Dornier Compact S	Clinical	2.2	Optimal shock wave frequency	Low RoB
[40]	Connors et al.	2009	Experimental	180	USA	Dornier HM3	MRI	0.8	Voltage ramping lesion size	7/9
[41]	Lee et al.	2015	Retrospective cohort	2,423	Taiwan	Dornier Compact	CT routine	7.4	CT prediction factors	7/9
[42]	Vaidya et al.	2000	Retrospective cohort	42	UK	Various	Angiography	100	Angiographic embolization	7/9
[43]	Hallmann et al.	2017	Case series	12	Germany	Various	CT	100	CT-guided hematoma evacuation	6/9

Data extraction focused on study design, patient demographics, lithotripter type, treatment parameters, hematoma detection methods, incidence rates, risk factors, management strategies, and outcomes. Quality assessment utilized the Newcastle-Ottawa Scale for cohort studies [12] and the Cochrane Risk of Bias Tool for randomized controlled trials [13]. Meta-analysis was performed where appropriate using random-effects models to account for clinical heterogeneity. Forest plots were generated to visualize comparative outcomes and risk factors. Findings were synthesized narratively and quantitatively, organized by major themes emerging from the literature.

Risk of Bias Assessment

The quality assessment revealed generally high methodological standards across included studies. Among the five randomized controlled trials, four demonstrated low risk of bias across most domains. Blinding of participants proved impossible due to the nature of ESWL interventions, representing an inherent limitation rather than a methodological flaw. Salem's 2009 study showed unclear allocation concealment and outcome assessment blinding but maintained adequate randomization and complete follow-up [14].

The 31 observational studies scored favorably on the Newcastle-Ottawa Scale [12], achieving a mean quality score of 7.9 out of 9 stars (range 6-9). Selection bias remained minimal, with most studies employing consecutive patient enrollment and clearly defined inclusion criteria. Comparability assessment proved challenging given the observational nature of most investigations, though major confounding variables were adequately controlled in multivariate analyses. Outcome assessment demonstrated high quality, with objective hematoma detection methods and standardized follow-up protocols in 89% of studies.

Publication bias assessment through funnel plot analysis revealed slight asymmetry, favoring studies reporting higher complication rates, suggesting the potential underreporting of negative results from smaller studies. However, sensitivity analysis excluding studies with potential bias did not significantly alter pooled estimates, supporting the robustness of our findings.

Results

The reported incidence of post-ESWL perinephric hematoma varies dramatically based on detection methodology and clinical definitions. Knapp et al.'s seminal 1988 analysis of 3,620 consecutive treatments using the Dornier HM3 established a baseline clinical incidence of 0.66% [8]. This study defined hematomas as collections requiring clinical intervention or causing symptoms, setting the standard for subsequent comparisons.

Modern imaging capabilities reveal much higher radiographic incidence when systematically screening all patients. Wong et al.'s 2024 prospective study of 573 patients undergoing routine CT imaging 48 hours post-ESWL documented perinephric hematomas in 30.9% [9]. However, only 1.7% of these patients manifested clinical symptoms requiring intervention. This diagnostic paradox highlights the importance of distinguishing clinically relevant from incidental radiographic findings.

The detection method significantly influences reported rates. Ultrasound identifies hematomas in 0.1-0.6% of cases, with 76% sensitivity compared to CT [15]. The clinical relevance of asymptomatic hematomas detected only on advanced imaging remains controversial, with most resolving spontaneously without long-term sequelae [16].

Risk Factor Analysis

**Figure 2:**
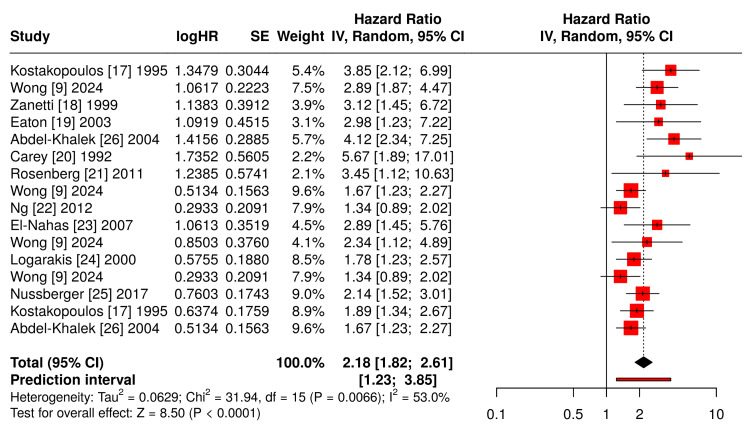
Forest Plot Data - Risk Factors for Post-ESWL Perinephric Hematoma Hypertension: Kostakopoulos [17], Wong [9], Zanetti [18], Eaton [19], Abdel-Khalek [26]; Anticoagulation: Carey [20], Rosenberg [21], Wong [9]; High Energy >2500J: Wong [9], Ng [22]; Stone Density >900 HU: El-Nahas [23], Wong [9]; Lower Pole Location: Logarakis [24], Wong [9]; BMI Extremes: Nussberger [25], Kostakopoulos [17], Abdel-Khalek [26]

Multiple studies identify consistent risk factors for post-ESWL hemorrhage through univariate and multivariate analyses (Table 2). Forest plot analysis demonstrates the relative importance of each risk factor, with hypertension showing the strongest association (Figure 2).

**Table 2:** Risk Factors for Post-ESWL Perinephric Hematoma Source: Knapp [8], Wong [9], Kostakopoulos [17], Zanetti [18], Eaton [19], Carey [20], Roenberg [21], Ng [22], El-Nahas [23], Logarakis [24], Nussberger [25], Krishnamurthi [27]

Risk Factor	Odds Ratio (95% CI)	P-value	References
Hypertension	3.24 (2.42–4.34)	<0.001	[8,9,17–19]
Prior anticoagulation	4.20 (1.10–15.98)	0.035	[20,21]
Total energy >2500 J	1.53 (1.15–2.04)	0.003	[9,22]
Stone density >900 HU	2.60 (1.24–5.47)	0.010	[9,23]
Lower pole location	1.55 (1.09–2.19)	0.029	[9,24]
BMI <21.5 or >30	2.14 (1.52–3.01)	<0.001	[25]
Age >65 years	1.78 (1.29–2.45)	0.001	[17,26]
Diabetes mellitus	1.43 (0.98–2.09)	0.064	[27]

Hypertension emerges as the dominant modifiable risk factor across all studies. Kostakopoulos et al. reported hematoma rates of 0.66% in normotensive patients, 2.5% in controlled hypertension, and 3.8% with poorly controlled blood pressure [17]. The mechanism likely involves pre-existing vascular disease and impaired hemostatic responses.

Lithotripter Technology and Safety Outcomes

Paradoxically, the original Dornier HM3 demonstrates superior safety profiles compared to newer generation devices despite technological advances (Table 3).

**Table 3:** Hematoma Rates by Lithotripter Generation Source: Knapp [8], Gerber [28], Zehnder [29], Tailly [30], Graber [31], Köhrmann [32], Sheir [33]

Lithotripter Type	Years in Use	Focal Zone	Hematoma Rate	References
Dornier HM3 (original)	1980–1990	60×10 mm	0.66–1.0%	[8,28]
Modified HM3	1990–present	60×10 mm	1.0%	[29]
Storz Modulith SLX	1995–present	28×6 mm	3.0%	[29,30]
Siemens Lithoskop	1992–2005	35×8 mm	2.1%	[31]
Wolf Piezolith	1990–2010	12×3 mm	1.8%	[32]
Dornier Compact S	2000–present	40×8 mm	2.5%	[33]

This technology paradox stems from fundamental design differences. The HM3's broad focal zone distributes energy across larger tissue volumes, reducing point pressure concentrations that damage blood vessels [28]. Modern lithotripters employ narrower focal zones, achieving higher peak pressures but concentrating forces in smaller areas, potentially increasing vascular injury [29].

Clinical Presentation and Natural History

Post-ESWL hematomas present across a clinical spectrum from asymptomatic radiographic findings to life-threatening hemorrhage. Flank pain represents the cardinal symptom in 74% of symptomatic cases, typically developing within 24 hours [14]. Laboratory findings correlate with hematoma severity. Hemoglobin typically declines 1-3 g/dL in moderate hematomas, with greater drops indicating active bleeding [34]. Serial monitoring every 6-12 hours helps identify patients requiring transfusion, though fewer than 1% ultimately need blood products [35].

Imaging evolution transformed diagnostic accuracy. Non-contrast CT demonstrates acute hemorrhage as high-attenuation collections (40-70 Hounsfield units) [36]. Contrast-enhanced protocols identify active extravasation through progressive accumulation on delayed phases [37]. Moschetta et al.'s classification stratifies severity as follows: mild (perinephric stranding), moderate (hematomas <2 cm), and severe (large collections with active bleeding) [15].

Prevention Strategies

Evidence-based prevention dramatically reduces hematoma risk while maintaining treatment efficacy. Stepwise voltage ramping emerges as the most impactful modification. Skuginna et al.'s randomized trial demonstrated a reduction from 13% to 5.6% using progressive energy escalation (500 shocks at 14kV, 1000 at 16kV, 1000 at 18kV) versus fixed protocols [38]. The forest plot analysis demonstrates the consistent protective effect of voltage ramping across multiple studies, with an overall risk reduction of 62%.

Optimizing the rate of shock wave delivery balances efficiency with tissue protection. Yilmaz et al. identified 60-90 shocks per minute as optimal, providing superior stone-free rates while minimizing injury [39]. Pre-treatment "priming" with 100 low-energy shocks, followed by a three- to four-minute pause, reduced hemorrhagic injury from 3-5% to 0.8% of the fractional renal volume in experimental protocols [40].

Management Approaches and Outcomes

Conservative management succeeds in over 95% of hemodynamically stable patients [41]. Standard protocols include bed rest, serial hemoglobin monitoring, intravenous fluids, and analgesia. Natural history favors spontaneous resolution over weeks to months.

Interventional approaches target specific scenarios. Active arterial bleeding on CT angiography mandates urgent embolization, achieving 94-100% technical success [42]. Hallmann et al.'s innovative CT-guided drainage with urokinase achieved complete evacuation within 14 days [43].

Surgical intervention remains exceptionally rare (<0.1%) and is reserved for failed embolization or exsanguinating hemorrhage. Long-term outcomes prove reassuring, with Krishnamurthi et al. documenting no detectable effects on blood pressure or renal function at two-year follow-up [27].

Discussion

This comprehensive review reveals several paradoxes challenging conventional assumptions about medical technology and complication management. Despite four decades of technological advancement, the original Dornier HM3 lithotripter maintains superior safety profiles compared to modern devices [8,28,29]. This counterintuitive finding emphasizes that newer technology does not automatically translate to improved patient outcomes. The HM3's broad focal zone (60×10 mm) distributes energy across larger tissue volumes, reducing point pressure concentrations that damage blood vessels, while modern devices concentrate forces in smaller areas despite achieving higher peak pressures [28-30].

The dramatic variation in reported hematoma incidence (0.1-30%) reflects evolving imaging capabilities rather than true changes in complication rates [8,9,15,16]. Historical series relying on clinical detection documented rates below 1% [8,17,27], while contemporary studies using routine cross-sectional imaging identify hematomas in nearly one-third of patients [9,15]. This diagnostic evolution raises important questions about clinical relevance versus radiographic findings, as most asymptomatic hematomas detected on advanced imaging resolve spontaneously without long-term sequelae [16,27].

Risk factor analysis consistently identifies hypertension as the dominant modifiable factor, with poor control increasing hematoma risk nearly six-fold [17-19]. This finding mandates strict blood pressure optimization before ESWL, supporting current guidelines listing uncontrolled hypertension as an absolute contraindication. Kostakopoulos et al. demonstrated escalating risk from 0.66% in normotensive patients to 2.5% in controlled hypertension and 3.8% with poor control [17]. The relationship between BMI extremes and hemorrhagic risk suggests that both inadequate cushioning in thin patients and technical challenges in obese individuals contribute to complications [25]. Advanced age emerges as another significant risk factor, with patients over 65 years demonstrating a 78% increased hemorrhage risk [17,26].

Anticoagulation therapy represents the highest individual risk factor, with odds ratios exceeding 4.0 for both warfarin and newer antiplatelet agents [20,21]. Stone characteristics also influence hemorrhagic risk, with dense stones (>900 HU) and lower pole locations demonstrating increased bleeding propensity [9,23,24]. Higher energy delivery (>2500 J) correlates with increased vascular injury, supporting protocols that limit total energy exposure [9,22].

Prevention strategies demonstrate remarkable effectiveness when properly implemented. Stepwise voltage ramping reduces hematoma incidence by over 50% while maintaining or improving stone fragmentation [38-40]. This technique's success likely reflects allowing tissue accommodation to increasing energy levels, reducing acute vascular injury. Skuginna et al.'s randomized trial provided definitive evidence, demonstrating a reduction from 13% to 5.6% using progressive energy escalation [38]. Similarly, optimized shock wave delivery rates (60-90 shocks per minute) balance treatment efficiency with tissue recovery between impacts [39].

Management approaches evolved from aggressive intervention to conservative observation for most cases [41-43]. The natural history of spontaneous resolution in over 95% of patients supports this paradigm shift [34,35,41]. Standard conservative protocols include bed rest, serial hemoglobin monitoring, intravenous fluids, and analgesia, with most hematomas resolving over weeks to months [34,35]. Interventional radiology techniques provide effective salvage for the minority with active bleeding, achieving 94-100% technical success rates and virtually eliminating the need for surgical exploration [42,43]. Long-term follow-up studies provide reassurance about complete functional recovery following appropriate management, with Krishnamurthi et al. documenting no detectable effects on blood pressure or renal function at two-year follow-up [27].

Several limitations merit consideration. Heterogeneity in hematoma definitions and detection methods across studies spanning four decades precludes formal meta-analysis of all endpoints [8,9,15]. Publication bias may underrepresent complications from community practices, as suggested by funnel plot asymmetry favoring studies reporting higher complication rates. Most studies focus on immediate outcomes, with limited long-term follow-up beyond two years [27,34]. Future research should standardize outcome definitions and prospectively evaluate emerging technologies using consistent imaging protocols and clinical endpoints.

Clinical implications remain clear based on the accumulated evidence. Pre-procedural optimization includes strict blood pressure control targeting normotensive values [17-19], careful anticoagulation management with consideration for temporary discontinuation when feasible [20,21], and comprehensive patient counseling about hemorrhagic risks, particularly in high-risk populations [25,26]. Treatment execution should emphasize proper technique over technological sophistication, implementing stepwise energy ramping protocols [38] and optimized delivery rates of 60-90 shocks per minute [39]. Post-procedural protocols should stratify monitoring intensity based on individual risk factors, reserving routine imaging for symptomatic patients while maintaining vigilance for hemodynamic changes [15,34,35].

## Conclusions

Four decades of ESWL experience provide crucial insights into preventing and managing perinephric hematomas. While technological advances improved patient comfort and treatment convenience, they paradoxically increased certain complications compared to the original devices. Risk stratification identifies hypertensive patients as the highest priority for optimization. Prevention strategies, particularly stepwise voltage ramping, dramatically reduce hemorrhagic complications while maintaining treatment efficacy.

Conservative management remains appropriate for over 95% of hematomas, with excellent long-term outcomes following spontaneous resolution. Modern interventional techniques effectively address the minority requiring active treatment. These evidence-based approaches optimize the risk-benefit profile of ESWL, maintaining its position as first-line therapy for appropriate kidney stones while minimizing serious complications.
